# Development of Silver-Containing Hydroxyapatite-Coated Antimicrobial Implants for Orthopaedic and Spinal Surgery

**DOI:** 10.3390/medicina58040519

**Published:** 2022-04-06

**Authors:** Tadatsugu Morimoto, Hirohito Hirata, Shuichi Eto, Akira Hashimoto, Sakumo Kii, Takaomi Kobayashi, Masatsugu Tsukamoto, Tomohito Yoshihara, Yu Toda, Masaaki Mawatari

**Affiliations:** Department of Orthopaedic Surgery, Faculty of Medicine, Saga University, 5-1-1 Nabeshima, Saga 849-8501, Japan; h.hirata.saga@gmail.com (H.H.); shu.1.uhs0306@gmail.com (S.E.); hassyhashimo@yahoo.co.jp (A.H.); sakumonkey76@yahoo.co.jp (S.K.); takaomi_920@yahoo.co.jp (T.K.); masa2goo99@yahoo.co.jp (M.T.); tomohito4113@yahoo.co.jp (T.Y.); darapon414@gmail.com (Y.T.); mawatam@cc.saga-u.ac.jp (M.M.)

**Keywords:** antimicrobial coated implants, safety, biocompatibility, osteoconductivity, silver, Ag-HA coating, iodine, antibiotics

## Abstract

The prevention of surgical site infections is directly related to the minimization of surgical invasiveness, and is in line with the concept of minimally invasive spine therapy (MIST). In recent years, the incidence of postoperative infections has been increasing due to the increased use of spinal implant surgery in patients at high risk of infection, including the elderly and easily infected hosts, the limitations of poor bone marrow transfer of antibiotics, and the potential for contamination of surgical gloves and instruments. Thus, the development of antimicrobial implants in orthopedic and spinal surgery is becoming more and more popular, and implants with proven antimicrobial, safety, and osteoconductive properties (i.e., silver, iodine, antibiotics) in vitro, in vivo, and in clinical trials have become available for clinical use. We have developed silver-containing hydroxyapatite (Ag-HA)-coated implants to prevent post-operative infection, and increase bone fusion capacity, and have successfully commercialized antibacterial implants for hip prostheses and spinal interbody cages. This narrative review overviews the present status of available surface coating technologies and materials; describes how the antimicrobial, safety, and biocompatibility (osteoconductivity) of Ag-HA-coated implants have been demonstrated for commercialization; and reviews the clinical use of antimicrobial implants in orthopedic and spinal surgery, including Ag-HA-coated implants that we have developed.

## 1. Introduction

Spinal implant infection is among the most common complications after spine surgery, with an overall reported incidence of 2–13% [1], despite major advances in prophylactic measures and aseptic surgery techniques. Infected cases are frequently difficult to treat, which causes a significant burden on the patient and surgeon, and a significant impact on the healthcare economy. Therefore, the prevention of surgical site infections is directly related to the minimization of the surgical invasiveness, and is in line with the concept of minimally invasive spine therapy (MIST).

As the population ages, more patients are at high risk for surgery for reasons such as osteoporosis, complications, and a weakened immune system [2]. In order to successfully prevent SSIs, it is essential to minimize the overall risk and bacterial load in high-risk patients intraoperatively [3]. Therefore, advanced technologies have led to the development of new materials and surface coatings that can prevent bacterial adhesion, kill bacteria, and destroy biofilms, which results in a reduction of the bacterial load both in terms of virulence and dosage. The “ideal” coating technology must meet the basic requirements for widespread clinical use, including antimicrobial resistance, safety, and osteoconductivity. In particular, antimicrobial materials (i.e., silver, iodine, and antibiotics) present problems of local and systemic biotoxicity, allergy, and resistance. As a matter of fact, silver, iodine, and antibiotics have all been reported to be potentially toxic to osteoblasts at high concentrations in vitro [4,5]. One solution to this problem is to adjust the concentration of the antimicrobial material, which means to adjust the concentration to make it less biotoxic and more antimicrobial. Another is to combine with materials that enhance osteoconductivity. In addition, cost, logistics, ease of use, intellectual property rights, and regulatory approvals remain barriers to productization and commercialization. Therefore, although antimicrobial implants for orthopedic use have been extensively studied, few have been applied clinically, and even fewer have been commercialized [4,5,6,7,8]. As far as the biotechnological issues of commercialization of antimicrobial implants are concerned, it is essential to understand the current state of the art of surface coating techniques and materials, and to prove their antimicrobial, safety, and biocompatibility (osteoconductivity).

**Figure 1:**
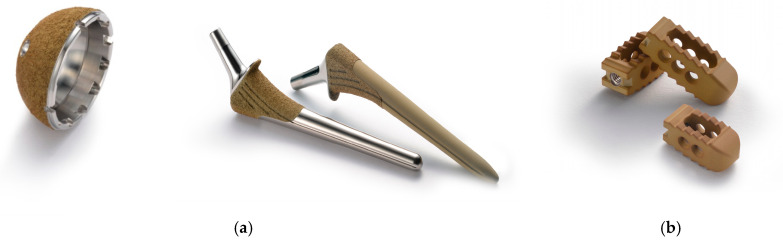
Silver-containing hydroxyapatite (Ag-HA) coating hip system (cup and stem) (**a**) and lumbar interbody cage (**b**).

We have produced a silver-containing hydroxyapatite (Ag-HA) coating by thermal spraying (Kyocera, Kyoto, Japan), which interfaces osteoconductive hydroxyapatite (HA) with antibacterial Ag [9,10]. Since Ag-HA coatings have been established to have good biocompatibility and low toxicity in vitro and in vivo, the technology has been applied to Ag-HA-coated implants for cementless prostheses with good clinical results without adverse events (Figure 1a) [11]. Then, an Ag-HA-coated lumbar interbody fusion cage (Ag-HA cage) was developed to prevent post-operative spinal implant infection, and increase fusion capacity, and the world’s first spinal antibacterial implant was successfully commercialized in 2020 (Resitage™, Kyocera) (Figure 1b).

In this paper, we apply a narrative review approach, and introduce various antimicrobial materials, technologies, and implants, and describe the Ag-HA-coated implants we have developed. In this narrative review, Section 2 overviews the present status of useful surface coating technologies and materials. Section 3 describes how the antimicrobial, safety, and biocompatibility (osteoconductivity) of Ag-HA-coated implants were demonstrated and commercialized, followed by a review of antimicrobial implants in clinical use in orthopedic and spinal surgery, including Ag-HA-coated implants.

## 2. Review of Antimicrobial Coatings Technologies and Materials

Implants used in orthopedic surgery must not be cytotoxic. Simultaneously, they must have an affinity with bone and soft tissue, which are adjacent to achieve mechanical stabilities. Regarding infection prevention for orthopedic implants, Gristina et al. created the concept of “race for the surface”, a term used to describe host cells and bacteria competing to adhere to the surface to govern the dominance [12]. Ideally, host cells will successfully “defend” the surface, preventing bacterial invasion and infection together with the host’s immune function [12]. Improving host immune function is not easy, and is highly individual-dependent. Therefore, a promising improvement in infection prevention is to develop new infection-resistant coatings. These also required not to be competitive with local cells or tissues. In addition to being biocompatible, they must be inexpensive and function to achieve their purpose at the site where they are placed, such as stimulating new bone formation; therefore, until now, there has been a lot of research focused specifically on osseointegration to develop ideal biomaterials in the orthopedic field. Four opportunities are available to us to prevent bacterial infection: (1) inhibition of bacterial adhesion; (2) inhibition of colony formation; (3) inhibition of biofilm formation; and (4) destruction of bacteria and the inhibition of bacterial growth [4,5,10,11]. Therefore, the strategies mainly inhibit adhesion, colonization, and biofilm formation. Coatings have been developed based on these factors.

Conventionally, implants have been classified into mainly two types: “passive” implants, which are coated to prevent bacterial adhesion; and “active” implants, which were created based on the idea of actively destroying bacteria by releasing substances from implants coated with antibiotics, and which show antibacterial activity [4,5,6,7,8]. In recent years, however, technological advancement has led to the development of coatings that are difficult to categorize, such as “contact killing,” which has a passive mechanism, but an active antimicrobial coating that destroys bacteria upon contact [13]. Thus, though precise classification has become very difficult, these two classifications are valid, and this paper will mainly introduce antimicrobial materials according to these two classifications.

### 2.1. Passive Surface Modification

The surface layer of existing implants could be chemically or physically processed to acquire antimicrobial properties. Examples include oxidation or mechanical modifications, such as roughening/polishing/texturing. Physical/chemical surface modifications, without the use of any pharmacologically active substance, can play a role in bacterial adhesion, proliferation, and, partially, bactericidal action as “contact killing.” Surface topography and roughness have a significant impact on the adhesion of bacteria to the material surface, which, in turn, has a considerable effect on the formation of biofilms. Hydrophobicity, electrostatic interactions, van der Waals forces, and steric hindrance have been reported to contribute to bacterial adhesion. Several studies have attempted to mimic the nanotexture of surfaces that exist in nature, such as cicada and dragonfly wings, lotus leaves, and sharkskin. In recent years, it has become possible to devise and fabricate ideal topographies that promote bone formation and inhibit bone resorption, and materials that inhibit bacterial adhesion and growth [13]. As a result, surface treatment nanotechnologies, such as nanopatterning, can provide new opportunities to develop effective anti-adhesion and antimicrobial treatments for orthopedic implants [14,15]. Thus, researchers are putting great emphasis on the development of materials with nanostructured surfaces that inhibit bacterial growth, biofilm formation, and, ultimately, bacterial infection, without side effects. Concerning chemical surface modifications, excellent anti-adhesion properties have also been reported. Further study is needed to determine the adverse side effects of these technologies, such as problems with mechanical properties, toxicity, and interference with osseointegration. Furthermore, only a few physical/chemical surface modifications appear suitable for clinical use. These new technologies’ in vivo efficacy and long-term effects on host cells and resistant bacteria are poorly understood. They need to be further investigated before clinical application and market introduction.

#### 2.1.1. Anti-Adhesion Polymers

If the density of the polymer is high enough, the polymer molecules are forced to stretch, and the resulting layer is called a “molecular brush”. The brush is essentially penetrable by solvents and low-molecular-weight ions; however, depending on its packing density, it may prevent the deposition of larger components, such as protein molecules and bacteria [16,17,18]. In addition to the antimicrobial effect of anti-adhesion by the spacer effect of the brush, antimicrobial peptides and Quaternary Ammonium Compounds (QAC) are added to the tip of the brush to perforate the cell membrane when bacteria adhere to it, resulting in bactericidal action [19,20,21].

#### 2.1.2. Albumin and Protein Coating

Albumin and proteins are believed to prevent bacteria from attaching to material through the principle of “surface competition”, and inhibition with bacterial cell adhesion factors. Albumin can also reduce bacterial adhesion by altering the hydrophobicity of a substrate surface [22,23]. Heparin can represent a specific inhibitor of the adhesion of *S. epidermidis* to biomaterials, which becomes coated with host fibronectin in vivo [24].

#### 2.1.3. TiO_2_

When TiO_2_ is irradiated with UV light, OH- is released around TiO_2_ by a photocatalytic reaction, which has an antibacterial effect [25]. UV-induced antimicrobial activity was confirmed, but to gain further versatility, the current research focuses on shifting the photocatalytic activity of such coatings towards the visible light range (e.g., by adding silver nanoparticles that can act through their surface plasmon resonance effects or molybdenum) [26,27].

### 2.2. Active Surface Modification

Materials with pharmacological bactericidal properties include antibiotics, antiseptics, metal ions (silver, copper, and others), non-metal elements (e.g., iodine, selenium), or organic substances (antibiotics, chitosan, other substances), and their combinations [5]. Moreover, various strategies, such as physical adsorption for coatings and chemical covalent conjugation for surface modifications, were applied to immobilize antimicrobials elements onto titanium surfaces. On the other hand, antimicrobial materials with pharmacological bactericidal effects have local and systemic toxicity, allergy, and resistance. Silver, iodine, and antibiotics have all been toxic to osteoblasts at high concentrations in vitro. These need special attention because it is desirable to promote bone formation around the implant, and maintain long-term osseointegration. Thus, achieving the optimal combination of antimicrobial effect and safety (or toxicity) is often a trade-off.

However, with promising technology on the horizon, it seems that the answer for reduced infection may lie in the synergy of many technologies. Next-generation coatings should be multifunctional, and integrate multiple antibacterial effects [28].

### 2.3. Antimicrobial Materials

Antimicrobial materials can be broadly classified into two major categories: (1) metals (e.g., silver, copper); and (2) non-metal elements (e.g., iodine, selenium) and organic substances (e.g., anti-infective peptides, chitosan) and their combinations. Typical materials are described in the text, and other materials with antimicrobial properties are briefly summarized in Table 1. Many substances have been reported to have antimicrobial activity, and their mechanisms (although many of them are not definite) have been reported.

There is a trade-off between toxicity to the human body and antimicrobial activity in all cases, and most require further research for commercialization.

#### 2.3.1. Metals

Ag and Cu are widely accepted metals. Ag, in particular, is the first material intentionally used in surgery because of its bactericidal properties [22]. In addition to Ag and Cu, Zn, Ni, Pb, Co, Mo, Zr, Cu have shown profound antimicrobial properties, reducing colony-forming units (CFU) of *E. coli* and *S. aureus*. Aside from their toxicity, Pb, followed by Co and Cu, have been reported to be the most effective materials against bacterial adhesion and growth [29]. On the other hand, Heidenau et al. [30] performed growth inhibition tests of several metal ions in the L929 cell line using several metal ions, and indicated that Ag and Zn ions were cytotoxic at low concentrations.

- Ag

Silver has long been used in the medical field. It has a broad antibacterial spectrum [9,10,11]. Furthermore, there have been no reports of resistant bacteria. Common complications of metal exposure, cytotoxicity, and human toxicity are feared at high concentrations; however, at low concentrations, the toxicity to osteoblasts and the effects on bone formation have been reported to be minimal [31]. Although the use of silver as a bulk material in medical devices is gradually declining, the use of various forms as a topical agent is common [31,32]. Due to its oligodynamic antibacterial activity, it shows bactericidal/antiseptic activity at very low concentrations, which results in a sustained and long-term effect [23]. These factors certainly make it the most used metal in wound care, and dental and orthopedic implant applications [33,34]. Furthermore, silver is contained in everyday items due to its expected antibacterial properties; thus, people are familiar with its usage.

- Cu

Development has progressed rapidly, and in recent years, copper has been widely used in the development of antimicrobial materials, with reviews published on copper-containing ceramics [35], copper-containing polymer composites [36], and copper-containing metal alloys [37]. It has been reported that copper has strong bactericidal properties, and can completely eliminate MRSA and *E. coli* [38,39]. High concentrations of Cu can cause growth inhibition, and are toxic to humans [17,18,19,20]. However, proper copper ions promote osteoblast proliferation, differentiation, and migration. Therefore, it is necessary to further investigate the appropriate concentration. 

#### 2.3.2. Non-Metal Elements

Non-metallic elements, such as hydrogen, chlorine, iodine, and oxygen, are commonly used in biomedicine because of their anti-infective properties. However, they have rarely been used as an antibacterial coating technology for orthopedic implants because they are generally soft and brittle [8]. Much research has been conducted, but it has not been commercialized at this time. In addition, implants permanently coated with antibiotics or other organic compounds that have never been used for local or systemic administration have ultimately been prevented from clinical application to date due to concerns about the development of resistant bacteria, toxicity, and the possibility of detrimental effects on the implant–bone union.

- Iodine

Iodine, one of the halogen elements, is an antibacterial substance that has long been a subject of research. It is widely used in the medical industry, from disinfecting surgical sites to gargling. Iodine is also the heaviest essential element needed by living organisms, and is a component of thyroid hormones. Titanium-iodine coating, which is produced electrically with a povidone-iodine electrolyte, is reported to have antibacterial activity [40].

**Table 1:** Antimicrobial materials.

	Antimicrobial Materials	Mechanism	Comments
Metals	Ag	(1) Destruction of cell walls and cytoplasmic membrane: silver ions (Ag+) released by silver nanoparticles adhere to or pass through the cell wall and cytoplasmic membrane. (2) Denaturation of ribosomes: silver ions degenerate ribosomes and inhibit protein synthesis. (3) Inhibition of adenosine triphosphate (ATP) production: ATP production is terminated because silver ions deactivate respiratory enzymes on the cytoplasmic membrane. (4) Membrane destruction by reactive oxygen species (ROS): ROS produced by the broken electron transport chain can cause membrane disruption. (5) Inhibition of deoxyribonucleic acid (DNA) replication: silver and reactive oxygen species bind to deoxyribonucleic acid, and prevent replication and cell multiplication. (6) Degeneration of membrane: silver nanoparticles accumulate in the cell wall pits, causing membrane degeneration. (7) Perforation of membrane: silver nanoparticles can migrate directly across the cytoplasmic membrane, and can release organelles from the cell [41].	A device for total hip arthroplasty coated with hydroxyapatite is now commercially available [11].
	Cu	Generation of ROS, lipid peroxidation, protein oxidation, and DNA degradation [42].	The U.S. Environmental Protection Agency certified copper as an antibacterial material in 2008 [6]
	Zn	Remains unclear. ROS generation and Zn ion release.	Non-cytotoxicity within a concentration from 10^−6^ M to 10^−5^ M [43,44].
	Ni	Four theories were proposed. (1) essential metals of metalloproteins are replaced by nickel; (2) nickel interrupts catalytic residues of non-metalloenzymes; (3) nickel allosterically inhibit enzymes by binding outside the catalytic site of them; and (4) nickel indirectly produces oxidative stress [45].	Ni^2+^as a dopant for ZnO. Used as Cu-Ni, Cu-Ni-Zn [46,47,48].
	Pb	Unclear.	Neurotoxicity is a matter of concern. Application to implants is difficult due to the problem of accumulation in the human body [49,50].
	Co	Unclear. Competitive inhibitor of iron during (Fe-S) synthesis in essential proteins for bacterial metabolism. [51].	Co has not been used as antibacterial materials and coatings so far [6].
	Mo, W	In situ production of H3Oþ ions by reacting with moisture from the air.	MoO3 has harmful effects on humans. However, it has been reported MoO3 processed into nanoparticles has low toxicity, the capability of biodegradation, and rapid excretion [52].
	Zr	Unclear. The interaction of positively-charged zirconium ions and negatively-charged cell wall [53].	ZrO2 nanoparticles are suggested as a potential antibacterial agent for Gram-negative bacteria.
	Ga	Inhibits bacterial metabolism.	Because the composition of gallium (III) is similar to that of iron (III), gallium competitively inhibits iron (III), and suppresses iron (III) function. [54].
	Ce	(1) Ce ions destroy cell walls and cell membranes because metal ions with strong reduction can extract electrons from the proteins of bacteria. (2) Ce ions can penetrate the cell and destroy the synzyme activity by reacting with the mercapto radical (–S.H.) (3) Ce ions can damage the enzyme system and normal metabolism of bacteria [55].	One of rare earth (RE). In practice, RE oxides and RE salts are commonly used with inorganic antimicrobial agents, such as TiO_2_, ZnO, Ag, Cu, and Zn.
	Sn	Changing the surface properties (wettability) to repel bacteria [56].	
	Sr	Inhibiting bacterial cytoplasmic membrane permeability, cell wall synthesis, bacterial chromosome replication, and cell metabolism.	Strontium facilitates bone formation by activating the calcium-sensing receptor, meanwhile inhibiting bone resorption by increasing osteoprotegerin, and preventing receptor activator of nuclear factor kappa B ligand expression [57,58].
	La	(1) La ions change the property of the cell wall. (2) La ions interrupt the normal physiological metabolism by interacting with DNA, enzymes, proteins, or other biological molecules, leading to the loss of Ca ions [59].	It has been reported that the concentration of around 0.15 wt.% La is considered to be the best trade-off.
non-Metals	Bacterial cell wall hydrolases	Degradation of cell wall, and impairment of cell wall synthesis.	Limitations against Gram-negative bacteria. Gram-positive pathogens have acquired resistance to lysozymes [60].
	Antimicrobial proteins peptides; AMPs	(1) Formation of ion channels or pores across the cytoplasmic membrane. (2) Inhibition of wall synthesis. (3) Activities of the ribonuclease (RNase) or deoxyribonuclease (DNase). (4) Depolarization and perforation of the cytoplasmic membrane [61].	A large family of peptides from diverse natural sources, having various structures and functionalities.
	Quaternary Ammonium Compounds; QAC	(1) Supporting biocides reach and perforate the cytoplasmic membrane.(2) Positively-charged QACs can detach phospholipids from the cell membrane [62,63,64].	It is practical to use polymer brushes as anchors, as it is with AMPs [20,21,65,66].
	Bacteriophages	Bacteriophages are viruses that infect bacteria.	It is relatively cost-effective. Bacteriophages are host-specific, but can infect several strains and species of bacteria, regardless of whether they are Gram-positive or Gram-negative. Immobilizing phage on sample surfaces such as gold, glass, cellulose membrane, and hydrogels was reported to exhibit antimicrobial activity [61,67,68,69]
	Fullerene	(1) oxidative stress production, (2) dysfunction of protein, (3) membrane injury, and (4) transcriptional arrest [70].	Fullerene is a closed-cage nanoparticle, where the conjugation is extended through π-electrons. Fullerenes generally produce a high rate of ROS by illumination.
	Carbon nanotubes; CNTs		Easily embedded into polymers. Synergistic effects were achieved by creating a CNTs–chitosan composite within the hydrogel, or by decorating CNTs with poly(amidoamine)dendrimer-immobilized carbon quantum dots or Ag2S quantum dots, which increased the antimicrobial activity in solution [71,72].
	Diamond-like carbon		Biofilm formation of *Pseudomonas aeruginosa* biofilm formation was significantly inhibited, but biofilms of Gram-positive *S. aureus* were ineffective [73].
	Graphene		Exhibits antibacterial activity in graphene, graphene oxide, and reduced graphene oxide. Synthesized from chitin, which is abundant in nature. Chitosan has a wide range of applications in medical fields, such as controlled drug delivery, wound dressing, tissue engineering, blood anticoagulant, bone regeneration biomaterial, and antimicrobial agent [70].
	Chitosan	It binds to negatively-charged bacterial cell walls, disrupting the cell and altering membrane permeability, then binds to DNA, inhibiting DNA replication and causing cell death.	Chitosan is a bioactive polymer with many applications due to its antimicrobial properties, non-toxicity, ease of modification, and biodegradability. [74,75].
	Plant extracts	Unclear.	Limited investigation has been conducted on its effectiveness on surfaces of healthcare units or on medical devices including tympanostomy tubes [76,77].
	Selenium	Unclear. Possibly free radical generation [78].	Antibacterial properties were also demonstrated by inhibiting the establishment of bacterial biofilms by *P. aeruginosa* and *S. aureus*. Selenium is a trace element in animal and human bodies [79,80].
	Acylase	Disruption of quorum sensing.	Acylase has been reported as a quorum quenching enzyme in Gram-negative bacteria [81].
	Chlorhexidine Chloroxylenol	Membrane disruption	Extensive applications in dentistry, such as gelatin for the treatment of periodontal infection, and in mouthwash [28,82,83].
	Octenidine	Perforation of the cytoplasmic membrane. Detachment of phospholipids from the cell membrane.	It has a wide spectrum of antimicrobial effectiveness against Gram-positive and Gram-negative bacteria and fungi [84].
	Cationic surfactants	(1) Membrane disruption after reaction with the cytoplasmic membrane (lipid or protein). (2) Leakage of intracellular low-molecular-weight substance. (3) Degradation of proteins and nucleic acids. (4) Wall perforation induced by autolytic enzymes.	Dioctadecyl dimethyl ammonium bromide (DODAB), hexadecyltrimethylammonium bromide (CTAB), and poly (diallyldimethyl) ammonium chloride (PDDA) are included [85].
	Nitric oxide	Disruption of cellular function and structure through interactions with microbial proteins, DNA, and metabolic enzymes.	NO reacts alone and with oxygen and reactive oxygen intermediates (e.g., superoxide and hydrogen peroxide) to form oxidative and nitrosative species, such as peroxynitrite RSNO, nitrogen dioxide, dinitrogen trioxide, and dinitrogen tetroxide, which exert nitrate-oxidative effects [86].
	Iodine	Perforate the cell wall, and disrupt protein and nucleic acid structure and synthesis [28,40].	Commercialization is problematic because it is difficult to adjust the dissolution speed, and ensure product uniformity. Chemical burn and irritant contact dermatitis cannot be overlooked.
	Chlorine	Destruction of cell walls and leakage of macromolecules by chlorination of substances in bacterial cell walls to produce chloro-compounds. [87].	It has long been widely used for disinfecting drinking water.
	Triclosan	Inhibition of fatty acid synthesis.	Triclosan acts as a biocide, targeting multiple cytoplasms and membranes at high concentrations [88,89,90].
	Furanones	Inhibition of quorum sensing.	Furanone compounds that inhibit bacterial quorum-sensing systems have been isolated from marine macro algae [91,92,93].

The concern with the use of metals is that in many cases, as the content increases, toxicity to host cells is observed. Therefore, the balance between antimicrobial activity and toxicity needs to be carefully monitored. Furthermore, some metals are clearly toxic, including lead (neurotoxic) and nickel (carcinogenicity) [6,45,49,50]. Therefore, the careful data accumulation of further data is required for commercialization. 

## 3. Review of Ag-HA Coated Antimicrobial Implants for Orthopedic and Spinal Surgery

### 3.1. Antimicrobial Efficacy of Antimicrobial Coatings and Materials

Evidence of antimicrobial efficacy has been investigated in terms of a broad spectrum, strong antimicrobial activity, prevention of cell adhesion, anti-biofilm effect, effective release kinetics (“peak effect”, i.e., large release of silver ions initially), long-lasting efficacy, low resistance, and synergy with antibiotics [6,94,95]. Several standard methods exist for testing the antimicrobial efficacy of materials in different countries and organizations [6]. Examples include the United State of America/ASTM G21-15, United Kingdom/BS ISO 22196: 2016, Japan/JIS Z 2801-2000, China/SN/T 2399- 2010, and ISO/ISO20645:2004 (country or organization/standard number) [1]. There are standard methods for evaluating the antimicrobial efficacy of materials in vitro, including plate counting, agar diffusion plate testing, confocal laser scanning microscopy (CLSM) in combination with fluorescent staining, and scanning electron microscopy (SEM) [6]. Previous studies have evaluated the antimicrobial efficacy of materials and coatings, such as Ag-HA coating, zinc-alloy, magnesium oxide coating, several metallic elements, iodine coating, and vancomycin coating, and were evaluated using the plate-count method, and/or CLSM, and/or SEM [10,40,95,96,97,98,99,100,101,102]. Sreekumari et al. [29] described resistance to the bacterial adhesion of various metals, such as Ni, Zn, Pb, Co, Mo, Zr, Cu, Sn, and Ti. With the exception of Sn and Ti, these metals showed good antibacterial activity toward *E. coli* and *Staphylococcus aureus*, and reduced the colony-forming units (CFU) from 10^6^ to less than 10^1^ within 24 h. We have demonstrated the antimicrobial properties of Ag-HA coatings in the following ways. First, we developed an Ag-HA coating method based on thermal spraying, and demonstrated silver ions were released from the Ag-HA coating in fetal bovine serum in vitro [9]. Then, we investigated the antibacterial and antibiofilm effects of Ag-HA coating in vitro [10,94,95]. Using the plate-count method, the Ag-HA coating was shown to have an antibacterial effect against *E. coli*, *Staphylococcus aureus,* and methicillin-resistant *Staphylococcus aureus (MRSA)*, whereas fluorescence microscopy, three-dimensional CLSM, and SEM demonstrated the antibiofilm effect against MRSA in vitro [10,94,95]. In addition, we demonstrated the time-dependent antibacterial and antibiofilm activity of the combination of Ag-HA and vancomycin with the plate-count method and three-dimensional CLSM in vitro [95].

In vivo studies of antibacterial materials have been highly disparate, and have not employed any standard method [6,103]. However, the assessment of the antibacterial effect of titanium-copper alloy in vivo was recently performed by common observation, leukocyte count test, plate-count method, and pathology [6,103,104]. In addition, the recommendation of the design and the antibacterial effects of materials in vivo was recently reported [103]. The study referred to model selection, study design, data interpretation, and targets for efficacy [104]. Past studies have also reported the antibacterial effect of Ag-HA or iodine coating in rabbit femur, using a pathological examination; vancomycin coating in mouse femur, using the plate-count method and X-ray imaging; and gentamicin coating in rabbit tibia, using blood tests, the plate-count method, and pathological examination [40,94,95,98,105,106,107,108,109,110,111]. Regarding other methods used to evaluate the antibacterial activity of materials in vivo, recent reports have used bioluminescent signals, which may be ethical and useful because the time-dependent assessment of antibacterial activity can be performed at regular intervals without euthanasia of animals [100,109,110]. We have also validated the efficacy of Ag-HA coating in vivo using several models and methods [94,95,105,106,107]. First, we reported the release of silver ions from Ag-HA coating using blood tests, and evaluated the antibacterial activity against MRSA using the plate-count method in a subcutaneous rat model [105]. Second, we demonstrated the released silver ions from the Ag-HA coating using blood tests, and the antibacterial activity toward MRSA in the medullary cavity of rat tibiae with the plate-count method, X-ray imaging, and pathological examination [106]. Third, using fluorescence microscopy, we found that the Ag-HA coating inhibited biofilm formation against MRSA, and the synergistic antibacterial effect of combining Ag-HA and vancomycin against MRSA using a plate-count method in a subcutaneous rat model [94,95]. In addition, we reported the antibacterial effectiveness of the Ag-HA coating at the medullary cavity of the rat femur against hematogenous infection with MRSA during the postoperative period using the plate-count method and blood tests [107]. 

### 3.2. Safety of Antimicrobial Coatings and Materials

Antimicrobial substances can be a double-edged sword, as they are antibacterial, but also biotoxic. Antimicrobial properties (i.e., silver, iodine, and antibiotics) have been reported to be potentially toxic to osteoblasts at high concentrations in vitro [1,4,5]. Several standard methods are used to access the cytotoxicity of materials in each country or organization, including Japan/JIST0993-1 and ISO/ISO 10993-5:2009 (country or organization/standard number). 

Test on extract, direct contact tests, and indirect contact tests (including agar diffusion or filter diffusion) have been mentioned as tests that are available for the evaluation of in vitro cytotoxicity (ISO 10993-5:2009). In previous studies, the cytotoxicity of materials and coatings, such as Ag-HA coating, iodine coating, gentamycin coating, and vancomycin coating, were reported using the test on extracts, and/or direct contact tests, and/or indirect contact tests [31,40,101,102,109,110,112,113,114,115,116]. We demonstrated the safety of Ag-HA coatings in the following way: we proved that the Ag-HA coating on the surface of orthopedic implants exhibited an antibacterial effect and inhibited bacterial adherence without cytotoxicity with the use of V79 Chinese hamster lung cells, which were found on Ag-HA coatings, as well as HA coatings in in vitro cytotoxicity studies [10].

First and foremost, in vivo cytotoxicity tests should be performed based on previous in vitro data, including the bioactive agent with and without any carrier [103]. Studies for local effects after implantation, and studies for systemic toxicity, are mentioned as typical studies for in vivo cytotoxicity (ISO 10993-6:2016, ISO 10993-11:2017). The past studies have reported no hepatotoxicity or nephrotoxicity of silver coating based on human blood tests and pathological examinations; no cytotoxicity or adverse effects of iodine coating in a clinical trial using iodine-supported titanium no nephrotoxicity or other side effects of gentamycin-coated implants; and no locally or systemically adverse events directly related to the fast-resorbable antibacterial hydrogel coating (DAC^®^, Novagenit Srl, Mezzolombardo, TN, Italy), which contains gentamycin, vancomycin, and meropenem [6,11,31,117,118,119,120]. 

We proved the safety and toxicity of the Ag-containing hydroxyapatite (Ag-HA) coating as shown below. First, we proved that the Ag-HA coating had in vivo antibacterial activities in rat tibia. Furthermore, we reported that the average concentration of Ag in serum reached a peak at approximately 48 h after implantation, at 3.3 ± 1.6 ppb, and then gradually decreased [106]. Wan et al. [121] noted that the normal human diet contains small amounts of silver, and since silver is consumed through an individual’s diet, blood silver concentrations below 200 ppb should be considered normal. Silver blood levels exceeding 300 ppb have been reported to cause argyria, and liver and kidney problems [9,10,11]. Therefore, the mean concentration of Ag in our report was low enough. Second, we demonstrated that in a model of rat tibia with Ag-HA-coated implants, the serum silver concentration was sufficiently low to have no detrimental effects, and that there was no degeneration in the brain, liver, kidney, or spleen [122]. The amount of silver required for Ag-HA coating of femoral replacements in humans is low enough to avoid argyria. Finally, we proved the safety of Ag-HA-coated implants in a prospectively interventional study [11]. We performed THA on 20 patients with this implant, and found that blood Ag levels peaked at 2 weeks after THA, and then gradually declined. The highest serum Ag concentration noted in postoperative follow-up was 6.0 ng/mL, which was in the normal range. Non-scientific reports emphasize the toxic effects of the release of silver ions from silver-coated implants, but both animal and human studies have shown that the blood silver levels never reached toxic levels [11]. There was no adverse reaction to Ag, and no argyria was observed. Furthermore, we conducted diagnostic imaging, and performed laboratory blood studies, including the measurement of leukocytes, hemoglobin, C-reactive protein (CRP), g-glutamyltransferase (GGT), glutamic-oxaloacetic transaminase (GOT), blood urea nitrogen (BUN), and creatinine, before and after surgery [11]. There was no evidence of implant failure or prosthetic joint infection at one year after surgery. No patients developed leukopenia, kidney damage, or liver damage [11].

### 3.3. The Biocompatibility of the Ag-HA

At the present time, orthopedic implants are mainly made of metals (cobalt chromium, stainless steel, and titanium). However, these metals often have no biological activity (e.g., osteoinduction). Therefore, coating materials that impart biological activity on the base metal have been developed [1]. Since orthopedic implants are inserted into the bone, they must have osteoinductivity, osteoconductivity, and osteointegration, in addition to being totally non-toxic [123,124]. In this section, we review the biocompatibility of Ag-HA with bone.

Osteoinductivity and osteoconductivity have been evaluated by in vitro studies. Osteoinductivity refers to the adhesion of undifferentiated stem cells from surrounding tissues or blood, and their differentiation into the osteogenic cell lineage, whereas osteoconductivity refers to the formation of bone on the surface of metals or coatings. These are examined by the differentiation and proliferation of cells spread on the metal or coating. Human and animal osteoblasts and osteoblast-like cells (MC3T3-E1, MG63, and SAOS-2) have been utilized in many studies [125,126,127]. Differentiation markers (e.g., osteocalcin, type-I-collagen, osteoprotegerin, glyceraldehyde-3-phosphate-dehydrogenase, and alkaline phosphatase (ALP)) have been adopted to evaluate differentiation into the osteogenic cell lineage [128,129]. We cultured MC3T3-E1, an osteoblast progenitor cell line, on Ag-HA-coated disks, and evaluated their differentiation into osteoblasts by measuring ALP [130]. The osteoblast cell line MC3T3-E1 cultivated on a 3% Ag-HA-coated surface showed no cytotoxicity, and production of alkaline phosphatase, an osteoblast marker, was observed. These results were consistent with those observed with silver-free HA coating. On the other hand, significantly higher cytotoxicity was demonstrated when the cells were cultivated on the 50% Ag-HA-coated surface [130].

In vivo studies have evaluated osteoconductivity and osseointegration. Metal implants (with or without coating) are inserted into the animal body, and bone formation at the surface of the substrate is assessed histologically. Dogs, rabbits, and rats are the animals that are most commonly employed [131,132,133]. If there is direct contact between the substrate and the bone tissue, it is assessed as possessing osteoconductivity. To quantify osteoconductivity, we measured and evaluated the affinity index, which is direct bone-to-substrate contact length divided by the total implant length, and multiplied by 100 [134].

Osseointegration refers to the mechanically strong connection of bone tissue in contact with a substrate. It requires mechanical evaluation, and is assessed by the shear stress of implants placed in animal bone. A push out test is often used for implants placed vertically in the femur of animals. In this case, the implant is fixed bi-cortically, which is suitable for the evaluation of screws and pins, but insufficient for the evaluation of implants in cancellous bone, such as those used in arthroplasty. We developed a model to evaluate anchorage strength by inserting the implant into the bone marrow of the femur, and pulling it out [130]. Since the implant was inserted into the bone marrow, it was possible to evaluate the histopathological characteristics of the implant by dividing it into two areas: the diaphyseal area (which is in contact with trabecular bone) and the metaphyseal area (which is in contact with cortical bone). This model was introduced at the Second International Consensus Meeting on Musculoskeletal Infections 2018 [135] as “Combining biomechanical and histological examination, the model of Eto et al. is valuable during the development phase of new anti-microbial implant surfaces to detect favourable solutions”.

### 3.4. Antimicrobial Implants for Clinical Use in Orthopaedic and Spinal Surgery

Research on antimicrobial orthopedic implants has been active, but few such implants have been applied clinically, and even fewer have been commercialized [4,5,8]. The potential toxicity associated with antimicrobial overdose (e.g., Ag and iodine) has limited the clinical application of antimicrobial implants. Table 2 shows a summary of antimicrobial implants that are available for clinical use, or at least for which clinical results have been reported, in the field of orthopedic and spinal surgery.

First, most reports of orthopedic antimicrobial implants have been for surgical operations using antimicrobial implants for extremity fractures [119,156] or antimicrobial megaprosthesis for bone tumor reconstruction surgery in the extremities, which is associated with a relatively high infection rate [6,31,136,137,138,139,140,141,142,143,144,145,146,147,148,149], with spinal application described in few cases [117,151,152,153,154]. Moreover, as for the type or site of the spinal antimicrobial instrumentation, the pedicle screw is expected based on the frequency of use and the intervertebral cage from the viewpoint of host-site immunity; however, most cases of clinical use described the use of antimicrobial pedicle screws. Secondly, with regard to antimicrobial materials for orthopedic antimicrobial implants, silver was reported most frequently, followed by iodine and antibiotics (gentamicin being the most commonly used) (Table 2).

Although not an antimicrobial implant, a fast-resorbable hydrogel coating that can be filled with a variety of antibacterial agents in the intraoperative setting has been successfully launched in the European market, and has been shown to prevent postoperative infection in orthopedic implants [120,157,158].

With regard to spinal antimicrobial implants, the use of silver and iodine pedicle screws and a rod system have been reported with good results. In addition, as already mentioned, after demonstrating the antibacterial activity, antibiofilm activity, osteoconductivity, and non-toxicity of the Ag-HA coating in vitro and in vivo, we first commercialized Ag-HA-coated implants for cementless THA (Ag-HA-coated hip system) in 2015 (Figure 1a), and Ag-HA cages (Resitage™) in 2021 (Figure 1b). A prospective multicenter clinical trial is currently ongoing (UMIN 000039964).

A meta-analysis demonstrated that antimicrobial coatings (e.g., silver, antibiotics, and iodine) are effective for reducing postoperative infection rates [159,160]. To our knowledge, in all reports, the reported silver and iodine levels in blood are very far from the threshold of toxicity, and no systemic complications have been reported in any study [11,117,159,160]. However, further large-scale clinical randomized controlled trials focusing on the antimicrobial properties and adverse events are considered necessary.

## 4. Conclusions

Antimicrobial measures are an essential part of MIST. In order to minimize the intraoperative bacterial load, orthopedic implants require not only osteoconductivity and safety, but also antimicrobial properties. Recently, various strategies for the placement of antimicrobial implants have been proposed, which suggests that the answer to reducing infections lies in the synergy of many technologies, not just one technique or material.

Although antimicrobial implants for orthopedic use have been extensively studied, few have been applied clinically, and even fewer have been commercialized. Throughout the world, economic, logistical, intellectual property, and high regulatory burdens are common barriers to the productization and commercialization of antimicrobial implants.

Most reports of antimicrobial implants in orthopedic surgery have been for fractures of extremities or bone tumor reconstruction surgery, with few reports of spinal antimicrobial implant surgery cases. Research into the development of future antimicrobial implants in the field of spinal surgery is warranted. In addition, a large-scale clinical trial of spinal antimicrobial implants focusing on antimicrobial resistance and adverse events will be required.

## Figures and Tables

**Table 2:** Antimicrobial implants for clinical use in orthopedic and spinal surgery.

Antimicrobial Material	Trademark (Company, Nationality)	Author	Regulatory Level	Coating Technology	Concentrations/Loading	Availability (Application)	Indications
Silver							
	Mutars^®^ (Implantcast, Germany)	Hardes [31,136,137], Glehr [6], Hussmann [138], Wilding [139], Piccioli [140], Donati [141], Zajonz [142], Schmolders [143], Trovarelli [144]	Market	Galvanic deposition of elementary silver on the gold layer	0.33–2.89 g	Upper and lower extremities (megaprosthesis)	Tumor
	Agluna^®^, METS^®^ (Stanmore Implants–Accentus Medical Ltd.,Oxford, UK)	Wafa [145], Medellin [146], Parry [147]	Market	Anodization of the titanium alloy, followed by absorption of silver from an aqueous solution	6 mg (maximum)	Upper and lower extremities (megaprosthesis)	Tumor
	PorAg^®^, Megasystem C ^®^ (Waldemar Link, Hamburg, Germany)	Scoccianti [148], Sambri [149]	Market	Silver plasma immersion ion implantation	Not specified	Upper and lower extremities (megaprosthesis)	Tumor
	AG-PROTEX^®^ Hip system (Kyocera, Kyoto, Japan)	Eto [11], Hashimoto [121], Kawano [150],	Market	Ag-HA was thermal sprayed as a coating material to fabricate an Ag-HA-coated implant	1.9 to 2.9-mg (hip system),	Lower extremities (hip prosthesis)	Hip osteoarthritis
	Resitage^®^ (Kyocera, Kyoto, Japan)	This report	Market	Ag-HA was thermal sprayed as a coating material to fabricate an Ag-HA-coated implant	0.1 to 0.8-mg (per cage)	Spine (lumbar interbody cage)	Lumar degenerative disease
	Not applicable (Turkey)	Seçinti [151]	Clinical	nanoparticle silver-coated implant	Not specified	Spine (pedicle screw and rod)	Spinal disease
Iodine							
	Not applicable (Japan)	Tsuchuya [117], Shirai [118], Demura [152], Hayashi [153], Kabata [154], Miwa [155]	Clinical	Povidone-iodine electrolyte-based process	10–12 μg/cm^2^	Upper and lower extremities/spine/ pelvis (prosthesis, nail, screw, plate)	Various cases (tumor, fracture, infection)
Gentamicin poly(D, L-lactide) matrix							
	UTN PROtect	Fuchs [119]	Market	Gentamicin poly (D, Llactide) with dip coating process	10–50 mg (per implant)	Lower extremities (Tibia nail)	Tibia fracture
	Expert Tibial Nail (ETN) PROtect	Metsemakers [156]	Market	Gentamicin poly (D, Llactide) with dip coating process	10–50 mg (per implant)	Lower extremities (Tibia nail)	Tibia fracture

## Data Availability

Not applicable.

## References

[1] McClelland S., Takemoto R.C., Lonner B.S., Andres T.M., Park J.J., Ricart-Hoffiz P.A., Bendo J.A., Goldstein J.A., Spivak J.M., Errico T.J. (2016). Analysis of Postoperative Thoracolumbar Spine Infections in a Prospective Randomized Controlled Trial Using the Centers for Disease Control Surgical Site Infection Criteria. Int. J. Spine Surg..

[2] Imajo Y., Taguchi T., Yone K., Okawa A., Otani K., Ogata T., Ozawa H., Shimada Y., Neo M., Iguchi T. (2015). Japanese 2011 nationwide survey on complications from spine surgery. J. Orthop. Sci..

[3] Yamada K., Abe H., Higashikawa A., Tonosu J., Kuniya T., Nakajima K., Fujii H., Niwa K., Shinozaki T., Watanabe K. (2018). Evidence-based Care Bundles for Preventing Surgical Site Infections in Spinal Instrumentation Surgery. Spine.

[4] Romanò C.L., Scarponi S., Gallazzi E., Romanò D., Drago L. (2015). Antibacterial coating of implants in orthopaedics and trauma: A classification proposal in an evolving panorama. J. Orthop. Surg. Res..

[5] Romanò C.L., Tsuchiya H., Morelli I., Battaglia A.G., Drago L. (2019). Antibacterial coating of implants: Are we missing something?. Bone Jt. Res..

[6] Zhang E., Zhao X., Hu J., Wang R., Fu S., Qin G. (2021). Antibacterial metals and alloys for potential biomedical implants. Bioact. Mater..

[7] Goodman S.B., Yao Z., Keeney M., Yang F. (2013). The future of biologic coatings for orthopaedic implants. Biomaterials.

[8] Gallo J., Holinka M., Moucha C. (2014). Antibacterial Surface Treatment for Orthopaedic Implants. Int. J. Mol. Sci..

[9] Noda I., Miyaji F., Ando Y., Miyamoto H., Shimazaki T., Yonekura Y., Miyazaki M., Mawatari M., Hotokebuchi T. (2009). Development of novel thermal sprayed antibacterial coating and evaluation of release properties of silver ions. J. Biomed. Mater. Res. B Appl. Biomater..

[10] Ando Y., Miyamoto H., Noda I., Miyaji F., Shimazaki T., Yonekura Y., Miyazaki M., Mawatari M., Hotokebuchi T. (2010). Effect of Bacterial Media on the Evaluation of the Antibacterial Activity of a Biomaterial Containing Inorganic Antibacterial Reagents or Antibiotics. Biocontrol Sci..

[11] Eto S., Kawano S., Someya S., Miyamoto H., Sonohata M., Mawatari M. (2016). First Clinical Experience with Thermal-Sprayed Silver Oxide-Containing Hydroxyapatite Coating Implant. J. Arthroplast..

[12] Gristina A.G. (1987). Biomaterial-centered infection: Microbial adhesion versus tissue integration. Science.

[13] Tsimbouri P.M., Fisher L., Holloway N., Sjostrom T., Nobbs A.H., Meek R.M.D., Su B., Dalby M.J. (2016). Osteogenic and bactericidal surfaces from hydrothermal titania nanowires on titanium substrates. Sci. Rep..

[14] Decuzzi P., Ferrari M. (2010). Modulating cellular adhesion through nanotopography. Biomaterials.

[15] Puckett S.D., Taylor E., Raimondo T., Webster T.J. (2010). The relationship between the nanostructure of titanium surfaces and bacterial attachment. Biomaterials.

[16] Kaper H.J., Busscher H.J., Norde W. (2003). Characterization of poly(ethylene oxide) brushes on glass surfaces and adhesion of *Staphylococcus epidermidis*. J. Biomater. Sci. Polym. Ed..

[17] Harris L.G., Tosatti S., Wieland M., Textor M., Richards R.G. (2004). *Staphylococcus aureus* adhesion to titanium oxide surfaces coated with non-functionalized and peptide-functionalized poly(L-lysine)-grafted-poly(ethylene glycol) copolymers. Biomaterials.

[18] Zhang F., Zhang Z., Zhu X., Kang E.T., Neoh K.G. (2008). Silk-functionalized titanium surfaces for enhancing osteoblast functions and reducing bacterial adhesion. Biomaterials.

[19] Muszanska A.K., Rochford E.T., Gruszka A., Bastian A.A., Busscher H.J., Norde W., van der Mei H.C., Herrmann A. (2014). Antiadhesive polymer brush coating functionalized with antimicrobial and RGD peptides to reduce biofilm formation and enhance tissue integration. Biomacromolecules.

[20] Yu K., Lo J.C., Yan M., Yang X., Brooks D.E., Hancock R.E., Lange D., Kizhakkedathu J.N. (2017). Anti-adhesive antimicrobial peptide coating prevents catheter associated infection in a mouse urinary infection model. Biomaterials.

[21] Gao G., Lange D., Hilpert K., Kindrachuk J., Zou Y., Cheng J.T., Kazemzadeh-Narbat M., Yu K., Wang R., Straus S.K. (2011). The biocompatibility and biofilm resistance of implant coatings based on hydrophilic polymer brushes conjugated with antimicrobial peptides. Biomaterials.

[22] Campoccia D., Montanaro L., Arciola C.R. (2013). A review of the biomaterials technologies for infection-resistant surfaces. Biomaterials.

[23] An Y.H., Bradley J., Powers D.L., Friedman R.J. (1997). The prevention of prosthetic infection using a cross-linked albumin coating in a rabbit model. J. Bone Jt. Surg. Br..

[24] Arciola C.R., Bustanji Y., Conti M., Campoccia D., Baldassarri L., Samorì B., Montanaro L. (2003). *Staphylococcus epidermidis*-fibronectin binding and its inhibition by heparin. Biomaterials.

[25] Hirakawa T., Yawata K., Nosaka Y. (2007). Photocatalytic reactivity for O_2_− and OH radical formation in anatase and rutile TiO_2_ suspension as the effect of H_2_O_2_ addition. Appl. Catal. A Gen..

[26] Fisher L., Ostovapour S., Kelly P., Whitehead K.A., Cooke K., Storgårds E., Verran J. (2014). Molybdenum doped titanium dioxide photocatalytic coatings for use as hygienic surfaces: The effect of soiling on antimicrobial activity. Biofouling.

[27] Dunnill C.W., Page K., Aiken Z.A., Noimark S., Hyett G., Kafizas A., Pratten J., Wilson M., Parkin I.P. (2011). Nanoparticulate silver coated-titania thin films—Photo-oxidative destruction of stearic acid under different light sources and antimicrobial effects under hospital lighting conditions. J. Photochem. Photobiol. A Chem..

[28] Cloutier M., Mantovani D., Rosei F. (2015). Antibacterial Coatings: Challenges, Perspectives, and Opportunities. Trends Biotechnol..

[29] Sreekumari K.R., Sato Y., Kikuchi Y. (2005). Antibacterial Metals—A Viable Solution for Bacterial Attachment and Microbiologically Influenced Corrosion. Mater. Trans..

[30] Heidenau F., Mittelmeier W., Detsch R., Haenle M., Stenzel F., Ziegler G., Gollwitzer H. (2005). A novel antibacterial titania coating: Metal ion toxicity and in vitro surface colonization. J. Mater. Sci. Mater. Med..

[31] Hardes J., Streitburger A., Ahrens H., Nusselt T., Gebert C., Winkelmann W., Battmann A., Gosheger G. (2007). The influence of elementary silver versus titanium on osteoblasts behaviour in vitro using human osteosarcoma cell lines. Sarcoma.

[32] Chopra I. (2007). The increasing use of silver-based products as antimicrobial agents: A useful development or a cause for concern?. J. Antimicrob. Chemother..

[33] Ansari A., Pervez S., Javed U., Abro M.I., Nawaz M.A., Qader S.A.U., Aman A. (2018). Characterization and interplay of bacteriocin and exopolysaccharide-mediated silver nanoparticles as an antibacterial agent. Int. J. Biol. Macromol..

[34] Riaz M., Zia R., Ijaz A., Hussain T., Mohsin M., Malik A. (2018). Synthesis of monophasic Ag doped hydroxyapatite and evaluation of antibacterial activity. Mater. Sci. Eng. C Mater. Biol. Appl..

[35] Kargozar S., Montazerian M., Hamzehlou S., Kim H.W., Baino F. (2018). Mesoporous bioactive glasses: Promising platforms for antibacterial strategies. Acta Biomater..

[36] Tamayo L., Azócar M., Kogan M., Riveros A., Páez M. (2016). Copper-polymer nanocomposites: An excellent and cost-effective biocide for use on antibacterial surfaces. Mater. Sci. Eng. C Mater. Biol. Appl..

[37] Wang P., Yuan Y., Xu K., Zhong H., Yang Y., Jin S., Yang K., Qi X. (2021). Biological applications of copper-containing materials. Bioact Mater..

[38] Noyce J.O., Michels H., Keevil C.W. (2006). Potential use of copper surfaces to reduce survival of epidemic meticillin-resistant *Staphylococcus aureus* in the healthcare environment. J. Hosp. Infect..

[39] Noyce J.O., Michels H., Keevil C.W. (2006). Use of copper cast alloys to control *Escherichia coli* O157 cross-contamination during food processing. Appl. Environ. Microbiol..

[40] Shirai T., Shimizu T., Ohtani K., Zen Y., Takaya M., Tsuchiya H. (2011). Antibacterial iodine-supported titanium implants. Acta Biomater..

[41] Yin I.X., Zhang J., Zhao I.S., Mei M.L., Li Q., Chu C.H. (2020). The Antibacterial Mechanism of Silver Nanoparticles and Its Application in Dentistry. Int. J. Nanomed..

[42] Chatterjee A.K., Chakraborty R., Basu T. (2014). Mechanism of antibacterial activity of copper nanoparticles. Nanotechnology.

[43] Hu H., Zhang W., Qiao Y., Jiang X., Liu X., Ding C. (2012). Antibacterial activity and increased bone marrow stem cell functions of Zn-incorporated TiO2 coatings on titanium. Acta Biomater..

[44] Bellanger X., Billard P., Schneider R., Balan L., Merlin C. (2015). Stability and toxicity of ZnO quantum dots: Interplay between nanoparticles and bacteria. J. Hazard. Mater..

[45] MacOmber L., Hausinger R.P. (2011). Mechanisms of nickel toxicity in microorganisms. Met. Integr. Biometal Sci..

[46] Eppakayala J., Mettu M.R., Pendyala V.R., Madireddy J.R. (2020). Synthesis, structural and optical properties of Ni doped ZnO nanoparticle—A chemical approach. Mater. Today Proc..

[47] Wilks S.A., Michels H., Keevil C.W. (2005). The survival of *Escherichia coli* O157 on a range of metal surfaces. Int. J. Food Microbiol..

[48] Naskar A., Lee S., Kim K.S. (2020). Antibacterial potential of Ni-doped zinc oxide nanostructure: Comparatively more effective against Gram-negative bacteria including multi-drug resistant strains. RSC Adv..

[49] Farmand F., Ehdaie A., Roberts C.K., Sindhu R.K. (2005). Lead-induced dysregulation of superoxide dismutases, catalase, glutathione peroxidase, and guanylate cyclase. Environ. Res..

[50] Naik M.M., Dubey S.K. (2013). Lead resistant bacteria: Lead resistance mechanisms, their applications in lead bioremediation and biomonitoring. Ecotoxicol. Environ. Saf..

[51] Ranquet C., Ollagnier-de-Choudens S., Loiseau L., Barras F., Fontecave M. (2007). Cobalt Stress in *Escherichia coli*: The effect on the iron-sulfur proteins. J. Biol. Chem..

[52] Piçarra S., Lopes E., Almeida P.L., de Lencastre H., Aires-de-Sousa M. (2019). Novel coating containing molybdenum oxide nanoparticles to reduce *Staphylococcus aureus* contamination on inanimate surfaces. PLoS ONE.

[53] Tabassum N., Kumar D., Verma D., Bohara R.A., Singh M.P. (2021). Zirconium oxide (ZrO2) nanoparticles from antibacterial activity to cytotoxicity: A next-generation of multifunctional nanoparticles. Mater. Today Commun..

[54] Kelson A.B., Carnevali M., Truong-Le V. (2013). Gallium-based anti-infectives: Targeting microbial iron-uptake mechanisms. Curr. Opin. Pharmacol..

[55] Jing H., Wu X., Liu Y., Lu M., Yang K., Yao Z., Ke W. (2007). Antibacterial property of Ce-bearing stainless steels. J. Mater. Sci..

[56] Verissimo N.C., Geilich B.M., Oliveira H.G., Caram R., Webster T.J. (2015). Reducing *Staphylococcus aureus* growth on Ti alloy nanostructured surfaces through the addition of Sn. J. Biomed. Mater. Res. Part A.

[57] Lin Y., Yang Z., Cheng J., Wang L. (2008). Synthesis, characterization and antibacterial property of strontium half and totally substituted hydroxyapatite nanoparticles. J. Wuhan Univ. Technol.-Mater. Sci Ed..

[58] Fielding G.A., Roy M., Bandyopadhyay A., Bose S. (2012). Antibacterial and biological characteristics of silver containing and strontium doped plasma sprayed hydroxyapatite coatings. Acta Biomater..

[59] Yuan J.P., Li W., Wang C. (2013). Effect of the La alloying addition on the antibacterial capability of 316L stainless steel. Mater. Sci. Eng. C.

[60] Parisien A., Allain B., Zhang J., Mandeville R., Lan C.Q. (2008). Novel alternatives to antibiotics: Bacteriophages, bacterial cell wall hydrolases, and antimicrobial peptides. J. Appl. Microbiol..

[61] Adlhart C., Verran J., Azevedo N.F., Olmez H., Keinänen-Toivola M.M., Gouveia I., Melo L.F., Crijnsg F. (2018). Surface modifications for antimicrobial effects in the healthcare setting: A critical overview. J. Hosp. Infect..

[62] Crismaru M., Asri L.A., Loontjens T.J., Krom B.P., de Vries J., van der Mei H.C., Busscher H.J. (2011). Survival of Adhering Staphylococci during Exposure to a Quaternary Ammonium Compound Evaluated by Using Atomic Force Microscopy Imaging. Antimicrob. Agents Chemother..

[63] Tiller J.C., Liao C.J., Lewis K., Klibanov A.M. (2001). Designing surfaces that kill bacteria on contact. Proc. Natl. Acad. Sci. USA.

[64] Murata H., Koepsel R.R., Matyjaszewski K., Russell A.J. (2007). Permanent, non-leaching antibacterial surfaces—2: How high density cationic surfaces kill bacterial cells. Biomaterials.

[65] Lewis K., Klibanov A.M. (2005). Surpassing nature: Rational design of sterile-surface materials. Trends Biotechnol..

[66] Fu W., Forster T., Mayer O., Curtin J.J., Lehman S.M., Donlan R.M. (2010). Bacteriophage cocktail for the prevention of biofilm formation by *Pseudomonas aeruginosa* on catheters in an in vitro model system. Antimicrob. Agents Chemother..

[67] Tawil N., Sacher E., Mandeville R., Meunier M. (2013). Strategies for the Immobilization of Bacteriophages on Gold Surfaces Monitored by Surface Plasmon Resonance and Surface Morphology. J. Phys. Chem. C.

[68] Hosseinidoust Z., van de Ven T.G.M., Tufenkji N. (2011). Bacterial capture efficiency and antimicrobial activity of phage-functionalized model surfaces. Langmuir.

[69] Anany H., Chen W., Pelton R., Griffiths M.W. (2011). Biocontrol of *Listeria monocytogenes* and *Escherichia coli* O157:H7 in meat by using phages immobilized on modified cellulose membranes. Appl. Environ. Microbiol..

[70] Rojas-Andrade M.D., Chata G., Rouholiman D., Liu J., Saltikov C., Chen S. (2017). Antibacterial mechanisms of graphene-based composite nanomaterials. Nanoscale.

[71] Neelgund G.M., Oki A., Luo Z. (2012). Antimicrobial activity of CdS and Ag2S quantum dots immobilized on poly(amidoamine) grafted carbon nanotubes. Colloids Surf. B Biointerfaces.

[72] Venkatesan J., Jayakumar R., Mohandas A., Bhatnagar I., Kim S.K. (2014). Antimicrobial Activity of Chitosan-Carbon Nanotube Hydrogels. Materials.

[73] Robertson S.N., Gibson D., MacKay W.G., Reid S., Williams C., Birney R. (2017). Investigation of the antimicrobial properties of modified multilayer diamond-like carbon coatings on 316 stainless steel. Surf. Coat. Technol..

[74] Muxika A., Etxabide A., Uranga J., Guerrero P., de la Caba K. (2017). Chitosan as a bioactive polymer: Processing, properties and applications. Int. J. Biol. Macromol..

[75] Yilmaz Atay H. (2020). Antibacterial Activity of Chitosan-Based Systems. Functional Chitosan.

[76] Kwieciński J., Eick S., Wójcik K. (2009). Effects of tea tree (*Melaleuca alternifolia*) oil on *Staphylococcus aureus* in biofilms and stationary growth phase. Int. J. Antimicrob. Agents.

[77] Ponce A.G., Roura S.I., del Valle C.E., Moreira M.R. (2008). Antimicrobial and antioxidant activities of edible coatings enriched with natural plant extracts: In vitro and in vivo studies. Postharvest Biol. Technol..

[78] Tran P.L., Hammond A.A., Mosley T., Cortez J., Gray T., Colmer-Hamood J.A., Reid T.W. (2009). Organoselenium coating on cellulose inhibits the formation of biofilms by *Pseudomonas aeruginosa* and *Staphylococcus aureus*. Appl. Environ. Microbiol..

[79] Rodríguez-Valencia C., Lopez-Álvarez M., Cochón-Cores B., Pereiro I., Serra J., González P. (2013). Novel selenium-doped hydroxyapatite coatings for biomedical applications. J. Biomed. Mater. Res. Part A.

[80] Tran P.A., Webster T.J. (2013). Antimicrobial selenium nanoparticle coatings on polymeric medical devices. Nanotechnology.

[81] Ivanova K., Fernandes M.M., Mendoza E., Tzanov T. (2015). Enzyme multilayer coatings inhibit *Pseudomonas aeruginosa* biofilm formation on urinary catheters. Appl. Microbiol. Biotechnol..

[82] Zhao L., Chu P.K., Zhang Y., Wu Z. (2009). Antibacterial coatings on titanium implants. J. Biomed. Mater. Res. Part B Appl. Biomater..

[83] Eltorai A.E.M., Haglin J., Perera S., Brea B.A., Ruttiman R., Garcia D.R., Born C.T., Daniels A.H. (2016). Antimicrobial technology in orthopedic and spinal implants. World J. Orthop..

[84] Baier G., Cavallaro A., Friedemann K., Müller B., Glasser G., Vasilev K., Landfester K. (2014). Enzymatic degradation of poly(L-lactide) nanoparticles followed by the release of octenidine and their bactericidal effects. Nanomed. Nanotechnol. Biol. Med..

[85] Carmona-Ribeiro A.M., de Melo Carrasco L.D. (2013). Cationic antimicrobial polymers and their assemblies. Int. J. Mol. Sci..

[86] Carpenter A.W., Schoenfisch M.H. (2012). Nitric Oxide Release Part, I.I. Therapeutic Applications. Chem. Soc. Rev..

[87] Kim J., Pitts B., Stewart P.S., Camper A., Yoon J. (2008). Comparison of the Antimicrobial Effects of Chlorine, Silver Ion, and Tobramycin on Biofilm. Antimicrob. Agents Chemother..

[88] Russell A.D. (2004). Whither triclosan?. J. Antimicrob. Chemother..

[89] Weber D.J., Rutala W.A. (2013). Self-disinfecting surfaces: Review of current methodologies and future prospects. Am. J. Infect. Control..

[90] Wang Z.X., Jiang C.P., Cao Y., Ding Y.T. (2013). Systematic review and meta-analysis of triclosan-coated sutures for the prevention of surgical-site infection. Br. J. Surg..

[91] Wu H., Song Z., Hentzer M., Andersen J.B., Molin S., Givskov M., Høiby N. (2004). Synthetic furanones inhibit quorum-sensing and enhance bacterial clearance in *Pseudomonas aeruginosa* lung infection in mice. J. Antimicrob. Chemother..

[92] Vasilev K., Cook J., Griesser H.J. (2009). Antibacterial surfaces for biomedical devices. Expert Rev. Med. Devices.

[93] Baveja J.K., Willcox M.D.P., Hume E.B.H., Kumar N., Odell R., Poole-Warren L.A. (2004). Furanones as potential anti-bacterial coatings on biomaterials. Biomaterials.

[94] Ueno M., Miyamoto H., Tsukamoto M., Eto S., Noda I., Shobuike T., Kobatake T., Sonohata M., Mawatari M. (2016). Silver-Containing Hydroxyapatite Coating Reduces Biofilm Formation by Methicillin-Resistant *Staphylococcus aureus* In Vitro and In Vivo. BioMed Res. Int..

[95] Hashimoto A., Miyamoto H., Kii S., Kobatake T., Shobuike T., Noda I., Sonohata M., Mawatari M. (2021). Time-dependent efficacy of combination of silver-containing hydroxyapatite coating and vancomycin on methicillin-resistant *Staphylococcus aureus* biofilm formation in vitro. BMC Res. Notes.

[96] García-Mintegui C., Córdoba L.C., Buxadera-Palomero J., Marquina A., Jiménez-Piqué E., Ginebra M.-P., Cortina J.L., Pegueroles M. (2021). Zn-Mg and Zn-Cu alloys for stenting applications: From nanoscale mechanical characterization to in vitro degradation and biocompatibility. Bioact. Mater..

[97] Coelho C.C., Padrão T., Costa L., Pinto M.T., Costa P.C., Domingues V.F., Quadros P.A., Monteiro F.J., Sousa S.R. (2020). The antibacterial and angiogenic effect of magnesium oxide in a hydroxyapatite bone substitute. Sci. Rep..

[98] Jennings J.A., Carpenter D.P., Troxel K.S., Beenken K.E., Smeltzer M.S., Courtney H.S., Haggard W.O. (2015). Novel Antibiotic-loaded Point-of-care Implant Coating Inhibits Biofilm. Clin. Orthop. Relat. Res..

[99] Tang T., Lin W.-T., Tan H.-L., Duan Z.-L., Yue B., Ma R., He G. (2014). Inhibited bacterial biofilm formation and improved osteogenic activity on gentamicin-loaded titania nanotubes with various diameters. Int. J. Nanomed..

[100] Honda M., Kawanobe Y., Ishii K., Konishi T., Mizumoto M., Kanzawa N., Matsumoto M., Aizawa M. (2013). In vitro and in vivo antimicrobial properties of silver-containing hydroxyapatite prepared via ultrasonic spray pyrolysis route. Mater. Sci. Eng. C.

[101] Ketonis C., Parvizi J., Adams C.S., Shapiro I.M., Hickok N.J. (2009). Topographic Features Retained after Antibiotic Modification of Ti Alloy Surfaces: Retention of Topography with Attachment of Antibiotics. Clin. Orthop. Relat. Res..

[102] Lawson M.C., Hoth K.C., Deforest C.A., Bowman C.N., Anseth K.S. (2010). Inhibition of *Staphylococcus epidermidis* biofilms using polymerizable vancomycin derivatives. Clin. Orthop Relat. Res..

[103] Moriarty T.F., Harris L.G., Mooney R.A., Wenke J.C., Riool M., Zaat S.A.J., Moter A., Schaer T.P., Khanna N., Kuehl R. (2019). Recommendations for design and conduct of preclinical in vivo studies of orthopedic device-related infection. J. Orthop. Res..

[104] Wang X., Dong H., Liu J., Qin G., Chen D., Zhang E. (2019). In vivo antibacterial property of Ti-Cu sintered alloy implant. Mater. Sci. Eng. C.

[105] Shimazaki T., Miyamoto H., Ando Y., Noda I., Yonekura Y., Kawano S., Miyazaki M., Mawatari M., Hotokebuchi T. (2009). In vivo antibacterial and silver-releasing properties of novel thermal sprayed silver-containing hydroxyapatite coating. J. Biomed. Mater. Res. Part B Appl. Biomater..

[106] Akiyama T., Miyamoto H., Yonekura Y., Tsukamoto M., Ando Y., Noda I., Sonohata M., Mawatari M. (2013). Silver oxide-containing hydroxyapatite coating has in vivo antibacterial activity in the rat tibia. J. Orthop. Res..

[107] Kobatake T., Miyamoto H., Hashimoto A., Ueno M., Nakashima T., Murakami T., Noda I., Shobuike T., Sonohata M., Mawatari M. (2019). Antibacterial Activity of Ag-Hydroxyapatite Coating Against Hematogenous Infection by Methicillin-Resistant *Staphylococcus aureus* in the Rat Femur. J. Orthop. Res..

[108] Liu D., He C., Liu Z., Xu W. (2017). Gentamicin coating of nanotubular anodized titanium implant reduces implant-related osteomyelitis and enhances bone biocompatibility in rabbits. Int. J. Nanomed..

[109] Stavrakis A.I., Zhu S., Loftin A.H., Weixian X., Niska J., Hegde V., Segura T., Bernthal N.M. (2019). Controlled Release of Vancomycin and Tigecycline from an Orthopaedic Implant Coating Prevents *Staphylococcus aureus* Infection in an Open Fracture Animal Model. Biomed. Res. Int..

[110] Funao H., Nagai S., Sasaki A., Hoshikawa T., Tsuji T., Okada Y., Koyasu S., Toyama Y., Nakamura M., Aizawa M. (2016). A novel hydroxyapatite film coated with ionic silver via inositol hexaphosphate chelation prevents implant-associated infection. Sci. Rep..

[111] Ishihama H., Ishii K., Nagai S., Kakinuma H., Sasaki A., Yoshioka K., Kuramoto T., Shiono Y., Funao H., Isogai N. (2021). An antibacterial coated polymer prevents biofilm formation and implant-associated infection. Sci. Rep..

[112] Coccini T., Manzo L., Bellotti V., De Simone U. (2014). Assessment of Cellular Responses after Short- and Long-Term Exposure to Silver Nanoparticles in Human Neuroblastoma (SH-SY5Y) and Astrocytoma (D384) Cells. Sci. World J..

[113] Hashimoto A., Sonohata M., Kitajima M., Kawano S., Eto S., Mawatari M. (2020). First experience with a thermal-sprayed silver oxide-containing hydroxyapatite coating implant in two-stage total hip arthroplasty for the treatment of septic arthritis with hip osteoarthritis: A case report. Int. J. Surg. Case Rep..

[114] Ketonis C., Barr S., Adams C.S., Shapiro I.M., Parvizi J., Hickok N.J. (2011). Vancomycin bonded to bone grafts prevents bacterial colonization. Antimicrob. Agents Chemother..

[115] Antoci V., King S.B., Jose B., Parvizi J., Zeiger A.R., Wickstrom E., Freeman T.A., Composto R.J., Ducheyne P., Shapiro I.M. (2007). Vancomycin covalently bonded to titanium alloy prevents bacterial colonization. J. Orthop. Res..

[116] Ketonis C., Adams C.S., Barr S., Aiyer A., Shapiro I.M., Parvizi J., Hickok N.J. (2010). Antibiotic Modification of Native Grafts: Improving Upon Nature’s Scaffolds. Tissue Eng. Part A.

[117] Tsuchiya H., Shirai T., Nishida H., Murakami H., Kabata T., Yamamoto N., Watanabe K., Nakase J. (2012). Innovative antimicrobial coating of titanium implants with iodine. J. Orthop. Sci..

[118] Shirai T., Tsuchiya H., Nishida H., Yamamoto N., Watanabe K., Nakase J., Terauchi R., Arai Y., Fujiwara H., Kubo T. (2014). Antimicrobial megaprostheses supported with iodine. J. Biomater. Appl..

[119] Fuchs T., Stange R., Schmidmaier G., Raschke M.J. (2011). The use of gentamicin-coated nails in the tibia: Preliminary results of a prospective study. Arch. Orthop. Trauma Surg..

[120] Zagra L., Gallazzi E., Romanò D., Scarponi S., Romanò C. (2019). Two-stage cementless hip revision for peri-prosthetic infection with an antibacterial hydrogel coating: Results of a comparative series. Int. Orthop..

[121] Wan A.T., Conyers R.A., Coombs C.J., Masterton J.P. (1991). Determination of silver in blood, urine, and tissues of volunteers and burn patients. Clin. Chem..

[122] Tsukamoto M., Miyamoto H., Ando Y., Noda I., Eto S., Akiyama T., Yonekura Y., Sonohata M., Mawatari M. (2014). Acute and Subacute ToxicityIn Vivoof Thermal-Sprayed Silver Containing Hydroxyapatite Coating in Rat Tibia. BioMed Res. Int..

[123] Chambers C., Proctor C., Kabler P. (1962). Bactericidal effect of low concentrations of silver. J. Am. Water Works Assoc..

[124] Albrektsson T., Johansson C. (2001). Osteoinduction, osteoconduction and osseointegration. Eur. Spine J..

[125] Shu R., McMullen R., Baumann M.J., McCabe L.R. (2003). Hydroxyapatite accelerates differentiation and suppresses growth of MC3T3-E1 osteoblasts. J. Biomed. Mater. Res. Part A.

[126] Mello A., Hong Z., Rossi A.M., Luan L., Farina M., Querido W., Eon J., Terra J., Balasundaram G., Webster T. (2007). Osteoblast proliferation on hydroxyapatite thin coatings produced by right angle magnetron sputtering. Biomed. Mater..

[127] Smith I.O., McCabe L.R., Baumann M.J. (2006). MC3T3-E1 osteoblast attachment and proliferation on porous hydroxyapatite scaffolds fabricated with nanophase powder. Int. J. Nanomed..

[128] Lim J.Y., Shaughnessy M.C., Zhou Z., Noh H., Vogler E.A., Donahue H.J. (2008). Surface energy effects on osteoblast spatial growth and mineralization. Biomaterials.

[129] Lai H.-C., Zhuang L.-F., Liu X., Wieland M., Zhang Z.-Y., Zhang Z.-Y. (2010). The influence of surface energy on early adherent events of osteoblast on titanium substrates. J. Biomed. Mater. Res. Part A.

[130] Eto S., Miyamoto H., Shobuike T., Noda I., Akiyama T., Tsukamoto M., Ueno M., Someya S., Kawano S., Sonohata M. (2015). Silver oxide-containing hydroxyapatite coating supports osteoblast function and enhances implant anchorage strength in rat femur. J. Orthop. Res..

[131] Søballe K. (1993). Hydroxyapatite ceramic coating for bone implant fixation. Mechanical and histological studies in dogs. Acta Orthop. Scand. Suppl..

[132] Chang B.-S., Lee I.C.K.F.C.-K., Hong K.-S., Youn H.-J., Ryu H.-S., Chung S.-S., Park K.-W. (2000). Osteoconduction at porous hydroxyapatite with various pore configurations. Biomaterials.

[133] Geesink R.G., de Groot K., Klein C.P. (1987). Chemical implant fixation using hydroxyl-apatite coatings. The development of a human total hip prosthesis for chemical fixation to bone using hydroxyl-apatite coatings on titanium substrates. Clin. Orthop. Relat. Res..

[134] Yonekura Y., Miyamoto H., Shimazaki T., Ando Y., Noda I., Mawatari M., Hotokebuchi T. (2011). Osteoconductivity of thermal-sprayed silver-containing hydroxyapatite coating in the rat tibia. J. Bone Jt. Surg. Br..

[135] Second International Consensus Meeting on Musculoskeletal Infection 2018. https://icmphilly.com/general-assembly/.

[136] Hardes J., Von Eiff C., Streitbuerger A., Balke M., Budny T., Henrichs M.P., Hauschild G., Ahrens H. (2010). Reduction of periprosthetic infection with silver-coated megaprostheses in patients with bone sarcoma. J. Surg. Oncol..

[137] Hardes J., Henrichs M.P., Hauschild G., Nottrott M., Guder W., Streitbuerger A. (2017). Silver-Coated Megaprosthesis of the Proximal Tibia in Patients with Sarcoma. J. Arthroplast..

[138] Hussmann B., Johann I., Kauther M.D., Landgraeber S., Jäger M., Lendemans S. (2013). Measurement of the Silver Ion Concentration in Wound Fluids after Implantation of Silver-Coated Megaprostheses: Correlation with the Clinical Outcome. Biomed. Res. Int..

[139] Wilding C.P., Cooper G.A., Freeman A.K., Parry M.C., Jeys L. (2016). Can a Silver-Coated Arthrodesis Implant Provide a Viable Alternative to Above Knee Amputation in the Unsalvageable, Infected Total Knee Arthroplasty?. J. Arthroplast..

[140] Piccioli A., Donati F., Giacomo G.D., Ziranu A., Careri S., Spinelli M.S., Giannini S., Giannicola G., Perisano C., Maccauro G. (2016). Infective complications in tumour endoprostheses implanted after pathological fracture of the limbs. Injury.

[141] Donati F., Di Giacomo G., D’Adamio S., Ziranu A., Careri S., Rosa M., Maccauro G. (2016). Silver-Coated Hip Megaprosthesis in Oncological Limb Savage Surgery. Biomed. Res. Int..

[142] Zajonz D., Birke U., Ghanem M., Prietzel T., Josten C., Roth A., Fakler J.K.M. (2017). Silver-coated modular Megaendoprostheses in salvage revision arthroplasty after periimplant infection with extensive bone loss—A pilot study of 34 patients. BMC Musculoskelet. Disord..

[143] Schmolders J., Koob S., Schepers P., Pennekamp P.H., Gravius S., Wirtz D.C., Placzek R., Strauss A.C. (2017). Lower limb reconstruction in tumor patients using modular silver-coated megaprostheses with regard to perimegaprosthetic joint infection: A case series, including 100 patients and review of the literature. Arch. Orthop. Trauma Surg..

[144] Trovarelli G., Cappellari A., Angelini A., Pala E., Ruggieri P. (2019). What Is the Survival and Function of Modular Reverse Total Shoulder Prostheses in Patients Undergoing Tumor Resections in Whom an Innervated Deltoid Muscle Can Be Preserved?. Clin. Orthop. Relat. Res..

[145] Wafa H., Grimer R.J., Reddy K., Jeys L., Abudu A., Carter S.R., Tillman R.M. (2015). Retrospective evaluation of the incidence of early periprosthetic infection with silver-treated endoprostheses in high-risk patients: Case-control study. Bone Jt. J..

[146] Medellin M.R., Fujiwara T., Clark R., Stevenson J.D., Parry M., Jeys L. (2019). Mechanisms of failure and survival of total femoral endoprosthetic replacements. Bone Jt. J..

[147] Parry M.C., Laitinen M.K., Albergo J.I., Gaston C.L., Stevenson J.D., Grimer R.J., Jeys L.M. (2019). Silver-coated (Agluna^®^) tumour prostheses can be a protective factor against infection in high risk failure patients. Eur. J. Surg. Oncol..

[148] Scoccianti G., Frenos F., Beltrami G., Campanacci D.A., Capanna R. (2016). Levels of silver ions in body fluids and clinical results in silver-coated megaprostheses after tumour, trauma or failed arthroplasty. Injury.

[149] Sambri A., Zucchini R., Giannini C., Zamparini E., Viale P., Donati D.M., De Paolis M. (2020). Silver-coated (PorAg^®^) endoprosthesis can be protective against reinfection in the treatment of tumor prostheses infection. Eur. J. Orthop. Surg. Traumatol..

[150] Kawano S., Sonohata M., Eto S., Kitajima M., Mawatari M. (2019). Bone ongrowth of a cementless silver oxide-containing hydroxyapatite-coated antibacterial acetabular socket. J. Orthop. Sci..

[151] Seçinti K.D., Attar A., Seçinti E. (2016). Clinical Trial Using A Silver-Coated Screw-Rod System and One-Year Follow-Up of The First 50 Patient. J. Nerv. Sys. Surg..

[152] Demura S., Murakami H., Shirai T., Kato S., Yoshioka K., Ota T., Ishii T., Igarashi T., Tsuchiya H. (2015). Surgical treatment for pyogenic vertebral osteomyelitis using iodine-supported spinal instruments: Initial case series of 14 patients. Eur. J. Clin. Microbiol. Infect. Dis..

[153] Hayashi H., Murakami H., Demura S., Kato S., Yoshioka K., Shinmura K., Yokogawa N., Ishii T., Fang X., Shirai T. (2015). Surgical site infection after total en bloc spondylectomy: Risk factors and the preventive new technology. Spine J..

[154] Kabata T., Maeda T., Kajino Y., Hasegawa K., Inoue D., Yamamoto T., Takagi T., Ohmori T., Tsuchiya H. (2015). Iodine-Supported Hip Implants: Short Term Clinical Results. Biomed. Res. Int..

[155] Miwa S., Shirai T., Yamamoto N., Hayashi K., Takeuchi A., Tada K., Kajino Y., Higuchi T., Abe K., Aiba H. (2019). Risk factors for surgical site infection after malignant bone tumor resection and reconstruction. BMC Cancer.

[156] Metsemakers W.J., Reul M., Nijs S. (2015). The use of gentamicin-coated nails in complex open tibia fracture and revision cases: A retrospective analysis of a single centre case series and review of the literature. Injury.

[157] Pitarresi G., Palumbo F.S., Calascibetta F., Fiorica C., Di Stefano M., Giammona G. (2013). Medicated hydrogels of hyaluronic acid derivatives for use in orthopedic field. Int. J. Pharm..

[158] Zoccali C., Scoccianti G., Biagini R., Daolio P.A., Giardina F.L., Campanacci D.A. (2021). Antibacterial hydrogel coating in joint mega-prosthesis: Results of a comparative series. Eur. J. Orthop. Surg. Traumatol..

[159] Savvidou O.D., Kaspiris A., Trikoupis I., Kakouratos G., Goumenos S., Melissaridou D., Papagelopoulos P.J. (2020). Efficacy of antimicrobial coated orthopaedic implants on the prevention of periprosthetic infections: A systematic review and meta-analysis. J. Bone Jt. Infect..

[160] Fiore M., Sambri A., Zucchini R., Giannini C., Donati D.M., De Paolis M. (2021). Silver-coated megaprosthesis in prevention and treatment of peri-prosthetic infections: A systematic review and meta-analysis about efficacy and toxicity in primary and revision surgery. Eur. J. Orthop. Surg. Traumatol..

